# Cellular and Immunohistochemical Changes in Anaphylactic Shock Induced in the Ovalbumin-Sensitized Wistar Rat Model

**DOI:** 10.3390/biom9030101

**Published:** 2019-03-13

**Authors:** Suhail Al-Salam, Elhadi H. Aburawi, Suleiman Al-Hammadi, Sekhar Dhanasekaran, Mohamed Shafiuallah, Javed Yasin, Manjusha Sudhadevi, Aktham Awwad, Seth L. Alper, Elsadig E. Kazzam, Abdelouahab Bellou

**Affiliations:** 1 Department of Pathology, College of Medicine & Health Sciences, United Arab Emirates University, AlAin, Abu Dhabi 17666, UAE; suhaila@uaeu.ac.ae (S.A.-S.); m.sudhadevi@uaeu.ac.ae (M.S.); 2 Department of Paediatrics, College of Medicine & Health Sciences, United Arab Emirates University, AlAin, Abu Dhabi 17666, UAE; E.aburawi@uaeu.ac.ae (E.H.A.); suleiman.alhammadi@uaeu.ac.ae (S.A.-H.); 3 Renaissance LLC, South Brunswick Dayton, New Jersey, NJ 08810, USA; sdsekar@outlook.com; 4 Department of Pharmacology, College of Medicine & Health Sciences, United Arab Emirates University, AlAin, Abu Dhabi 17666, UAE; m.shafiullah@uaeu.ac.ae; 5 Department of Internal Medicine, College of Medicine & Health Sciences, United Arab Emirates University, AlAin, Abu Dhabi 17666, UAE; javed.yasin@uaeu.ac.ae (J.Y.); e.kazzam@hotmail.com (E.E.K.); 6 Department of Laboratory Medicine, Tawam Hospital, AlAin, Abu Dhabi 15258, UAE; aadnan@seha.ae; 7 Division of Nephrology and Vascular Biology Research Center, Beth Israel Deaconess Medical Center, Department of Medicine, Harvard Medical School, Boston, MA 02215, USA; salper@bidmc.harvard.edu; 8 Department of Emergency Medicine, Beth Israel Deaconess Medical Center, Harvard Medical School, Boston, MA 02215, USA; 9 Global HealthCare Network & Research Innovation Institute, Brookline, MA 02446, USA; 10 International Board of Medicine and Surgery, Tampa, FL 34677, USA

**Keywords:** anaphylactic shock, cellular changes, tryptase, c-kit, iNOS, eNOS

## Abstract

Anaphylactic shock (AS) is a life-threatening, multisystem disorder arising from sudden release of mast cell- and basophil-derived mediators into the circulation. In this study, we have used a Wistar rat model to investigate AS-associated histopathologic changes in various organs. Rats were sensitized with ovalbumin (1 mg s.c), and AS was induced by intravenous injection of ovalbumin (1 mg). Experimental groups included nonallergic rats (*n* = 6) and allergic rats (*n* = 6). Heart rate and blood pressure were monitored during one hour. Organs were harvested at the end of the experiment and prepared for histologic and immunohistochemical studies. Lung, small bowel mucosa and spleen were found to undergo heavy infiltration by mast cells and eosinophils, with less prominent mast cell infiltration of cardiac tissue. The mast cells in lung, small bowel and spleen exhibited increased expression of tryptase, c-kit and induced nitric oxide synthase (iNOS). Increased expression of endothelial nitric oxide synthase (eNOS) by vascular endothelial cells was noted principally in lung, heart and small bowel wall. The Wistar rat model of AS exhibited accumulation of mast cells and eosinophils in the lung, small bowel, and spleen to a greater extent than in the heart. We conclude that lung and gut are principal inflammatory targets in AS, and likely contribute to the severe hypotension of AS. Targeting nitric oxide (NO) production may help reduce AS mortality.

## 1. Introduction

“Anaphylaxis” was recognized as a clinical syndrome in 1901 by Richet and Portier [1,2]. Anaphylactic shock (AS) is a multisystem disorder arising from sudden release into the circulation of potent mast cell- and basophil-derived inflammatory and vasodilatory mediators [3,4,5,6].

The majority of AS cases arise from type 1 hypersensitivity reaction to foods, medications, and insect stings [3]. AS is characterized by an abrupt release of mast cell- and basophil-derived mediators, including histamine, tryptase, serotonin, platelet-activating factor, leukotrienes, prostaglandins, cytokines, and nitric oxide (NO). The role of each of these mediators in the pathogenesis of AS remains incompletely defined [7,8]. Some reports have suggested that AS increases NO generation which can prolong the hypotension [9,10]. The three isoforms of nitric oxide synthase (NOS) are encoded by different genes and differ in function and tissue distribution. Brain NOS and endothelial NOS (eNOS) are constitutively expressed and under tight regulation so as to generate limiting amounts of NO that are rapidly metabolized. In contrast, inducible NOS (iNOS) can be transcriptionally upregulated by cytokines or endotoxins to generate rapid increases in local levels of NO [11].

The few pathological studies available are derived from autopsies of patients who died following AS [12]. In this study, we have used a Wistar rat model of AS to study AS-associated cellular changes in various organs, in which cellular expression of tryptase, c-KIT, iNOS and eNOS was assessed by immunohistochemistry.

## 2. Results

### 2.1. Blood Pressure and Heart Rate After Induction of AS with Ovalbumin Challenge

Mean arterial pressure (MAP) and heart rate (HR) in controls were stable throughout the 60 min duration of the experiment. In AS rats, MAP decreased by 66% within 5 min post-ovalbumin challenge, and by 87% within 30 min (Supplementary Figure S1). 

### 2.2. Histological Evaluation of H&E-Stained Tissue Sections

#### 2.2.1. Lungs

**Figure 1:**
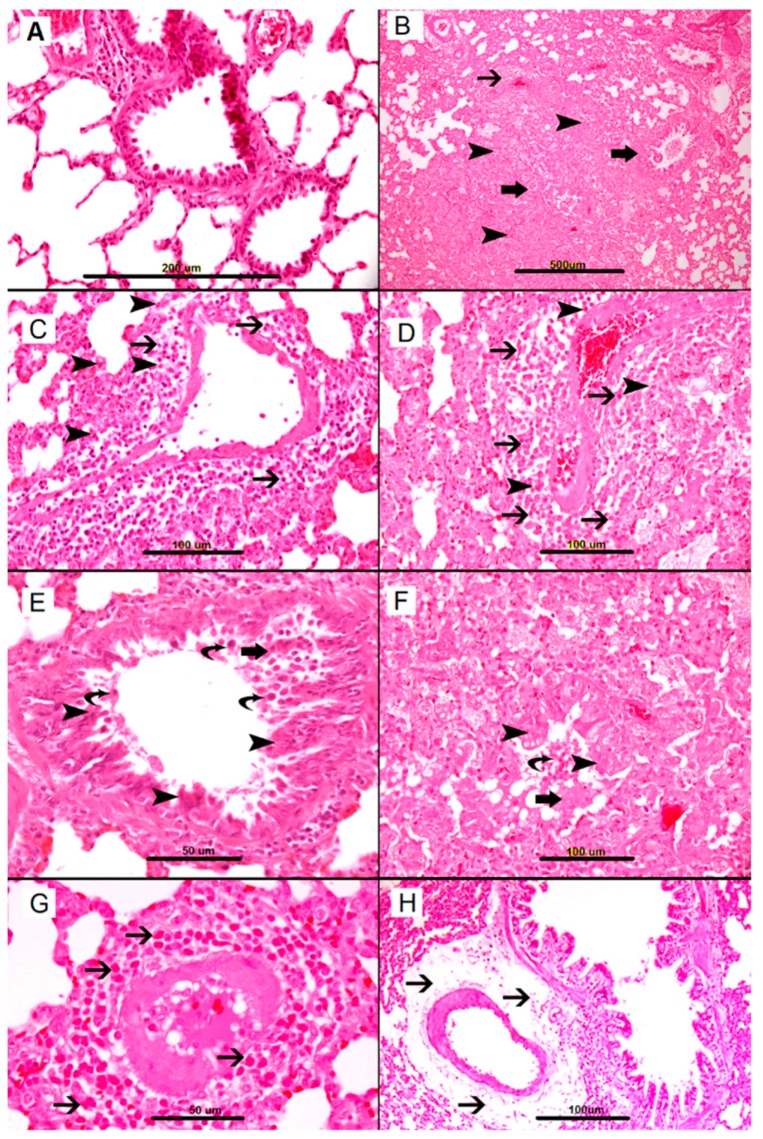
Representative sections of lung tissue. (**A**) Control group, showing lung tissue with patent alveolar spaces and bronchial passages and unremarkable blood vessels. (**B**–**H**) show anaphylactic changes in the lung; (**B**) Heavy mixed inflammatory cell infiltration of lung parenchyma with widening of interalveolar spaces (arrowheads), perivascular cellular infiltrates (thin arrows), and peribronchial inflammation (thick arrows). (**C**,**D**) Perivascular edema and heavy inflammatory cell infiltrate consisting predominantly of mast cells (thin arrows) and eosinophils (arrowheads). (**E**,**F**) Narrowing of the bronchial lumen with sloughing of respiratory epithelium (thick arrow), epithelial injury (arrowhead), and fallen dead cells in the lumen (curved arrow) (**G**) Heavy perivascular eosinophil infiltration (thin arrow), (**H**) Severe perivascular edema (thin arrow).

The nonallergic control group lungs exhibited normal architecture with patent air spaces and unremarkable interalveolar spaces. The bronchial passages were patent and appeared normal (Figure 1A). In contrast, lungs from the AS group exhibited abnormal architecture (Figure 1B–H). Alveolar air spaces were severely decreased due to widening of interalveolar spaces by inflammatory cell infiltration and edema. 

Distal airway passages were narrowed by mucosal edema and sloughing of epithelial cells into the lumen. Many detached respiratory epithelial cells were evident in the bronchial lumens (Figure 1E,F). Heavy peribronchial infiltration of inflammatory cells was evident, including mast cells, eosinophils, lymphocytes, macrophages and neutrophils. The perivascular inflammatory cell infiltration consisted predominantly of mast cells and eosinophils (Figure 1C,D,G). 

A prominent perivascular edema was present (Figure 1H). 

#### 2.2.2. Heart

**Figure 2:**
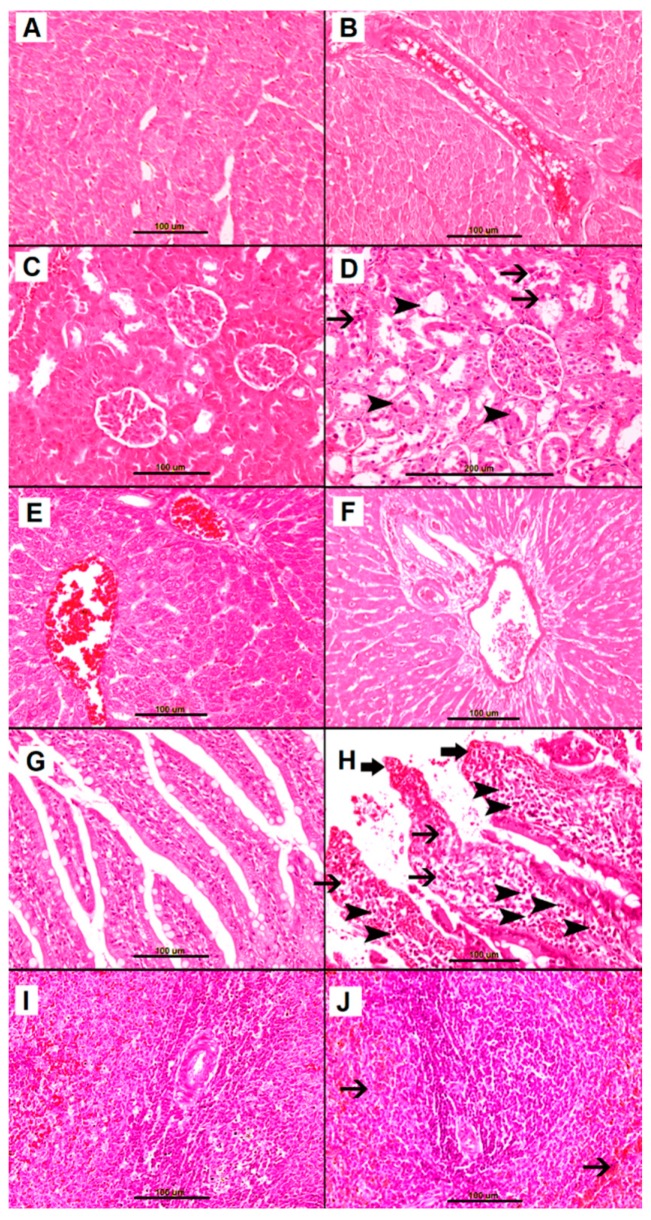
Representative sections of saline-treated naïve control group organs (**A**,**C**,**E**,**G**,**I**) and of anaphylactic shock (AS) organs (**B**,**D**,**F**,**H**,**J**). (**A**) Normal cardiac myocytes and blood vessels. (**C**) Normal kidney tissue with unremarkable tubules and glomeruli. (**E**) Normal liver tissue with unremarkable hepatocytes, central vein and portal tracts. (**G**) Normal finger-like small intestinal villi with preserved brush border and few mononuclear cellular infiltrates. (**I**) Normal spleen with unremarkable white and red pulp. (**B**) AS heart with unremarkable cardiac myocytes and blood vessels. (**D**) AS kidney tissue with acute tubular injury (arrowhead) and intraluminal fallen dead cells (thin arrow). (**F**) AS liver tissue with unremarkable hepatocytes, central vein and portal tract. (**H**) AS small bowel mucosa with disturbance of normal architecture, edematous widening of villi, and heavy mononuclear cell infiltration consisting predominantly of mast cells (arrowhead) and eosinophils (thin arrow), with sloughing of lining epithelium and deterioration of brush border (thick arrow). K. AS spleen with expansion of red pulp (thin arrow).

Control group hearts were normal, with unremarkable cardiac myocytes. The cardiac blood vessels were unremarkable (Figure 2A). Similarly, the AS group hearts were similarly normal with unremarkable cardiac myocytes and cardiac blood vessels. (Figure 2B).

#### 2.2.3. Kidney

Control group kidneys were normal, with unremarkable cortex and medulla, and unremarkable glomeruli and renal tubules (Figure 2C). In contrast, AS group kidneys showed features of acute tubular injury, including loss of brush border, tubular dilatation, shedding of cells into tubular lumen, and intratubular protein secretion (Figure 2D), without evident glomerular abnormality. 

#### 2.2.4. Livers

The livers of both control and AS groups were unremarkable, with unremarkable hepatocytes, central veins and portal tracts (Figure 2E,F). 

#### 2.2.5. Small Bowel

Control group small bowels were normal, with finger-like villi and unremarkable brush borders. (Figure 2G). In contrast, AS group small bowels were abnormal, with widening of the villi secondary to edema, and with heavy mononuclear cell infiltration consisting predominantly of mast cells. In addition, there was sloughing of surface epithelium and morphological deterioration of brush border (Figure 2H).

#### 2.2.6. Spleen

Control group spleens were normal, with unremarkable white pulp with central arteriole and red pulp (Figure 2I). AS group spleens were remarkable for marked expansion of the red pulp (Figure 2J)

### 2.3. Sirius Red Staining for Eosinophils

**Figure 3:**
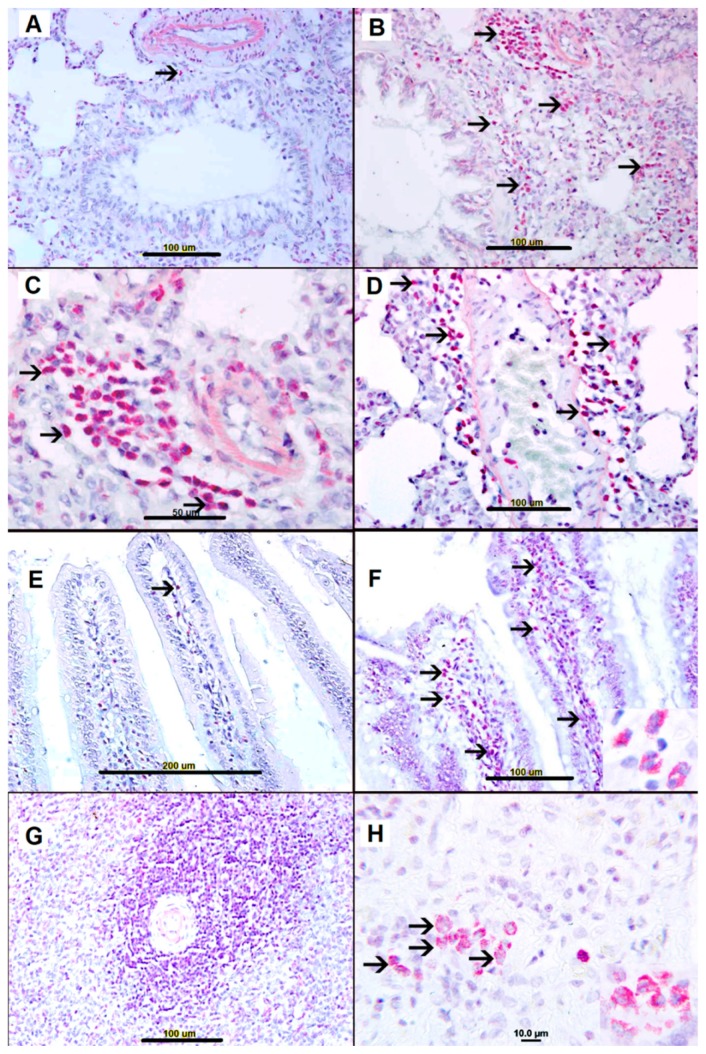
(**A**) Lung tissue from saline-treated naïve group showing unremarkable lung tissue with one perivascular eosinophil (arrow). (**B**–**D**) Representative sections of AS lung tissue showing heavy eosinophil infiltration in perivascular peribronchial and interstitial spaces (arrows). (**E**) Small bowel from saline-treated naïve group showing unremarkable villi with few eosinophils (arrow). (**F**) Representative sections of AS small bowel villi showing numerous eosinophils (arrows). (**G**) Unremarkable spleen tissue from saline-treated naïve group. (**H**) Representative sections of AS spleen showing numerous eosinophils (arrows).

Few eosinophils were evident in nonallergic control group lungs (Figure 3,A), small bowel villi (Figure 3E), and spleen (Figure 3G). Eosinophils were not seen in heart (Supplementary Figure S2A), kidney (Supplementary Figure S2C), and liver (Supplementary Figure S2E) in nonallergic control. In contrast, AS group lungs revealed heavy infiltration of eosinophils and polymorphic neutrophils, mainly in peribronchial (Figure 3B) and perivascular spaces (Figure 3C,D). Few eosinophils were seen in heart (Supplementary Figure S2B), kidney (Supplementary Figure S2D), and liver (Supplementary Figure S2F).

Heavy infiltration with eosinophils and polymorphic neutrophils was also evident in the small bowel villi (Figure 3F) and the splenic red pulp (Figure 3H). Heart, liver, and kidney sections from the AS group showed very few eosinophils (not shown). 

**Figure 4:**
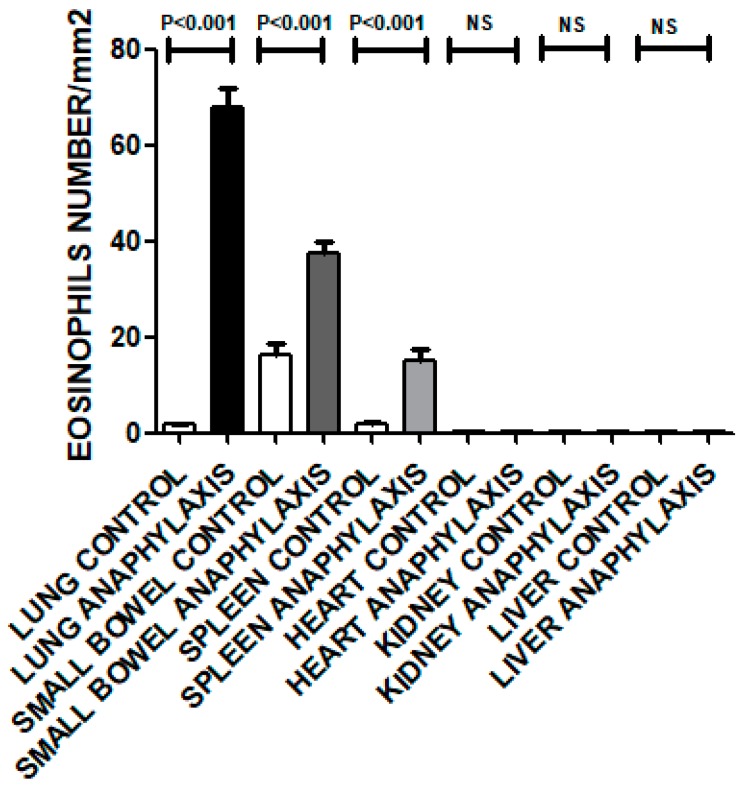
Morphometric analysis of eosinophils in lung, small bowel, spleen, heart, liver and kidney sections of the AS group as compared with the nonallergic control group. *p* < 0.05 is considered significant. NS: Not significant.

Morphometric analysis of Sirius Red-stained sections revealed a significant increase in the number of eosinophils in AS lungs (*p* < 0.001), small bowel (*p* < 0.001), and spleen (*p* < 0.001) as compared with their nonallergic control sections (Figure 4). In contrast, sections from heart, kidney, and liver exhibited nonsignificant differences in eosinophil numbers compared with their nonallergic controls (Figure 4).

### 2.4. Immunohistochemistry Studies

#### 2.4.1. Tryptase Expression

**Figure 5:**
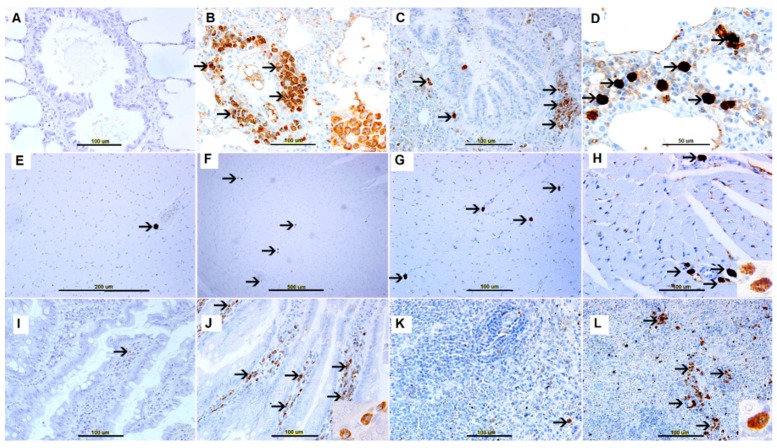
(**A**–**D**), Lung. (**A**) Unremarkable lung tissue from saline-treated nonallergic group. (**B**–**D**) Representative sections of AS lung tissue showing numerous tryptase-positive mast cells (arrows) in the (**B**) perivascular space, (**C**) peribronchial space, and (**D**) interstitial space. (**E**–**H**), Heart. (**E**) Unremarkable heart tissue from saline-treated nonallergic group showing one perivascular mast cell (arrow). (**F**–**H**) Representative sections of AS heart tissue showing scattered, perivascular tryptase-expressing mast cells (arrows). (**I**) Unremarkable small intestinal villi with one tryptase-positive mast cell (arrow). (**J**) Representative sections of AS small bowel villi showing many tryptase-positive mast cells (arrows). (**K**) Unremarkable spleen tissue from saline-treated nonallergic group showing one tryptase-positive mast cell (arrow). (**L**) Representative sections of AS splenic tissue showing many tryptase-expressing mast cells (arrows).

A few weakly tryptase-positive mast cells were evident in nonallergic control group lungs (Figure 5A), heart (Figure 5E), small bowel villi (Figure 5I), spleen (Figure 5K). Tryptase-positive mast cells were not identified in the kidney (Supplementary Figure S3A), and liver (Supplementary Figure S3C) in the nonallergic control group. In contrast, AS lungs 45 min post-AS induction were characterized by heavy infiltration of strongly tryptase-positive mast cells in perivascular (Figure 5B), peribronchial (Figure 5C) and interstitial spaces (Figure 5D). 

Anaphylactic shock hearts also revealed mast cells in the perivascular space (Figure 5F–H) and, to a much lesser extent, between cardiac myocytes and in the adventitia. In addition, heavy mast cell infiltration was evident in small bowel villi (Figure 5J) and splenic red pulp (Figure 5L). In contrast, AS group kidney and liver sections showed few mast cells (data not shown). Few Tryptase-positive mast cells were not identified in the kidney (Supplementary Figure S3B), and liver (Supplementary Figure S3D) in the nonallergic control group.

**Figure 6:**
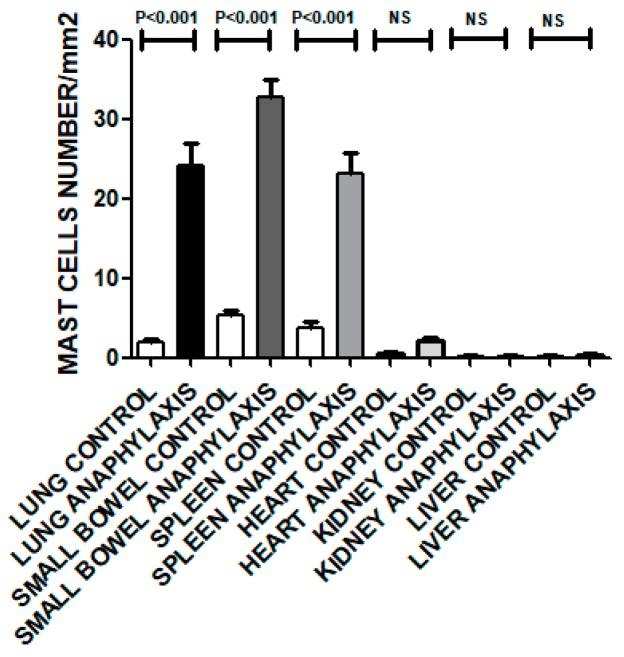
Morphometric analysis of mast cells in lung, small bowel, spleen, heart, liver and kidney sections of the AS group, compared with the nonallergic control group. *p* < 0.05 is considered significant. NS: Not significant.

Morphometric analysis of tryptase-positive cells revealed significantly increased numbers of mast cells in AS sections from lungs (*p* < 0.001), small bowel (*p* < 0.001), and spleen (*p* < 0.001), as compared with their nonallergic control sections (Figure 6).

In contrast, tryptase-stained sections from hearts, kidneys, and livers exhibited nonsignificant differences in mast cell numbers compared with nonallergic control sections (Figure 6).

#### 2.4.2. c-KIT Staining

**Figure 7:**
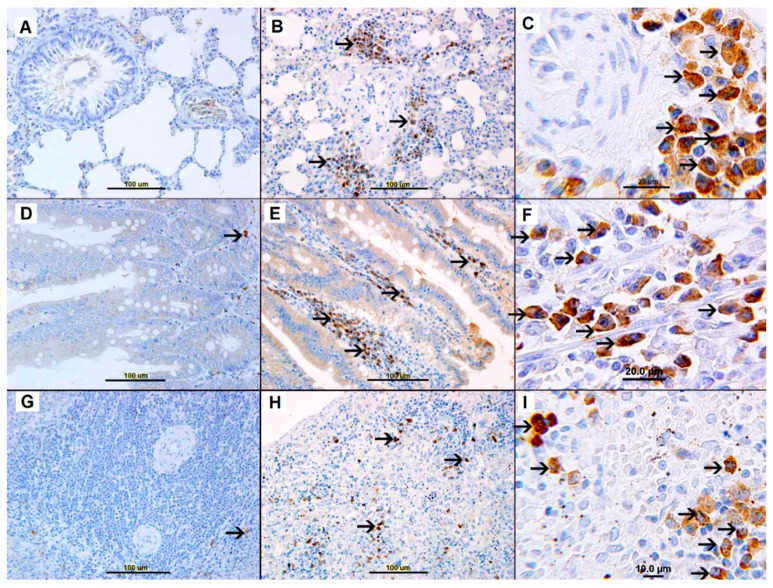
(**A**) Unremarkable lung tissue from saline-treated naïve group. (**B**,**C**) Representative section of AS lung tissue showing high expression of c-kit in mast cells (arrows) in perivascular and interstitial space. (**D**) Unremarkable small bowel villi with a single c-kit-positive mast cell (arrow) from saline-treated naïve group. (**E**,**F**) Representative sections of AS small bowel villi showing many c-kit-positive mast cells (arrows). (**G**) Unremarkable spleen tissue with c-kit-positive mast cells (arrows) from saline-treated naïve group. (**H**,**I**) Representative sections of splenic tissue during AS showing numerous c-kit-positive mast cells expressing c-kit (arrows).

A few c-kit-positive mast cells were present in sections from lung (Figure 7A), small bowel villi (Figure 7D), spleen (Figure 7G) of the nonallergic control group. C-KIT-positive mast cells were not identified in the heart (Supplementary Figure S4A), kidney (Supplementary Figure S4C) and liver (Supplementary Figure S4E) in the nonallergic control group. By 45 min post-AS induction, AS lungs were heavily infiltrated with c-kit-positive perivascular and interstitial mast cells (Figure 7B,C).

Heavy infiltration with c-kit-positive mast cells was also evident in sections of AS small bowel villi (Figure 7E,F) and splenic red pulp (Figure 7H,I). In contrast, AS sections from heart, kidney, and liver showed few mast cells (Supplementary Figure S4B,D,F). 

#### 2.4.3. iNOS Staining

**Figure 8:**
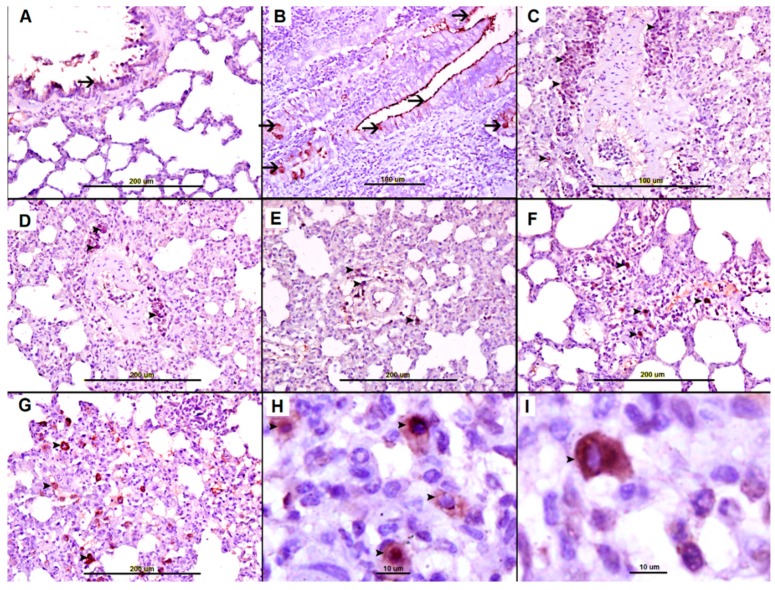
(**A**) Unremarkable lung tissue from saline-treated naïve group showing mild cytoplasmic iNOS expression by bronchial lining epithelium (arrows). (**B**–**I**), representative sections from AS lung tissue showing high cytoplasmic iNOS expression in (**B**) bronchial epithelium and goblet cells (arrows), (**C**–**E**) perivascular mast cells (arrowheads), (**F**) interstitial mast cells (arrowheads) and (**G**) macrophages (arrowheads). (**H**,**I**) show iNOS-expressing mast cells (arrowheads) at higher magnification.

Nonallergic control group bronchial epithelial cells showed low cytoplasmic iNOS expression (Figure 8A). iNOS expression was below detection threshold in spleen, kidney, heart, liver, and small bowel in the nonallergic control group (Supplementary Figure S5A,C,E,G,I). In contrast, 45 min post-AS induction, iNOS was highly expressed in AS sections of bronchial epithelial cells and goblet cells of lung (Figure 8B), as well as in perivascular and interstitial mononuclear cells consisting predominantly of mast cells (Figure 8C–F,H,I), and macrophages (Figure 8G). In addition, iNOS was expressed in macrophages in AS sections of spleen (Supplementary Figure S5B). Interestingly, iNOS expression was below detection threshold in kidney, heart, liver, and small bowel in the AS group (Supplementary Figure S5D,F,H,J). 

#### 2.4.4. eNOS Staining 

**Figure 9:**
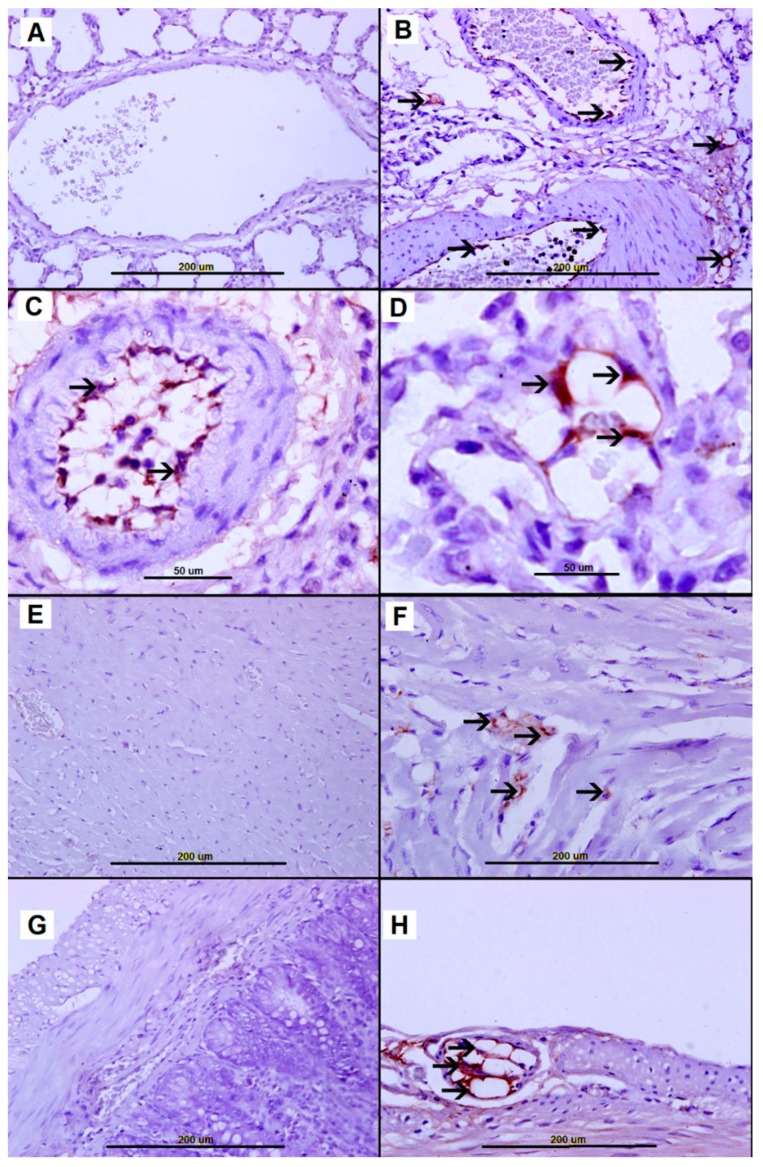
(**A**,**E**,**G**) eNOS-negative vascular endothelial cells from saline-treated naïve group lung (**A**), Heart (**E**), and small bowel (**G**). (**B**–**D)** Representative sections from AS lung tissue with elevated cytoplasmic eNOS expression in vascular endothelial cells (arrows). (**F**) Representative section from AS heart showing high cytoplasmic eNOS expression by vascular endothelial cells (arrows). (**H**) Representative section from AS small bowel wall showing high cytoplasmic NOS in vascular endothelial cells (arrow).

Cytoplasmic eNOS expression in endothelial cells from nonallergic control rats was below detection in lungs (Figure 9A), heart (Figure 9E), small bowel (Figure 9G), liver (Supplementary Figure S6A), kidney (Supplementary Figure S6C) and spleen (Supplementary Figure S6E). However, 45 min post-AS induction, eNOS expression was greatly elevated in vascular endothelial cells of AS lung (Figure 9B–D), heart (Figure 9F), and small bowel wall (Figure 9H). Interestingly, eNOS expression in endothelial cells of liver (Supplementary Figure S6B), kidney (Supplementary Figure S6D) and spleen (Supplementary Figure S6F) of the AS group was below the detection threshold.

## 3. Discussion

In AS, death usually results from severe hypotension and bronchoconstriction. Tissue hypoxia resulting from the severe hypotension and respiratory obstruction can lead to cardiac standstill and death [6]. 

In our model, acute IV challenge with ovalbumin induced severe hypotension and bradycardia, leading to the death of all AS group rats. This model replicates many cardiovascular physiological aspects of AS observed in humans [6,13,14], allowing exploration of clinically relevant pathophysiologic AS mechanisms. Characterization of the histopathologic changes that occur during AS in our model will help relate these changes to clinical presentation, and help eventually to elucidate AS mechanisms. 

The main histopathologic changes are seen in the lung. The lung parenchyma is heavily infiltrated by inflammatory cells, mainly mast cells, eosinophils, basophils, and lymphocytes, with less numerous macrophages and neutrophil polymorphs. It is unlikely that all these cells migrate to the lung during the 45 min following anaphylaxis. Some of these cells likely migrate to the lung during the two-week pre-AS sensitization period. 

The highly immunogenic OVA-adjuvant suspension leads to T lymphocyte activation and cytokine secretion that activates B lymphocytes to produce IgE, leading to mast cell sensitization. In addition, activated T lymphocytes will produce cytokines and growth factors that activate eosinophils, neutrophil polymorphs and macrophages [15]. Additional infiltrating cells evident in lung tissue of the AS group, especially mast cells and eosinophils, most likely migrate to the lung following induction of AS. Mast cells and eosinophils have been found to be increased in the respiratory system following allergic reaction [16,17]. The post-AS cellular response infiltrates interalveolar spaces and decreases or obliterates intra-alveolar spaces, resulting in hypoxemia due to decreased surface area for oxygen exchange. The peribronchial and intrabronchial cellular infiltrates, bronchoconstriction, luminal accumulation of sloughed epithelium, and mucosal and submucosa edema are associated with luminal narrowing. Similar findings have been reported by Grigoraș et al. [7]. 

Activation of the numerous perivascular mast cells evident in lung can release their granular contents into the systemic circulation to evoke the clinical features of AS. We have stained tissue sections with antitryptase and anti-c-kit antibodies to confirm and evaluate intensity of mast cell infiltration in lungs, small bowel and spleen. We found significantly higher numbers of tryptase-positive mast cells in AS group lungs (*p* < 0.001), small bowel (*p* < 0.001) and spleens (*p* < 0.001) than in the corresponding nonallergic control group organs (Figure 5 and Figure 6), findings consistent with those of Grigoras et al. [7], Unkrig et al. [18] and Pumphrey & Roberts [19].

We also used Sirius red stain to identify eosinophils in different organs. We found significantly higher numbers of Sirius Red-stained eosinophils in AS group lungs (*p* < 0.001), small bowel (*p* < 0.001) and spleens (*p* < 0.001) than in the corresponding organs of the nonallergic control group (Figure 4). This finding may reflect activated T cell and mast cell production of the strong eosinophil growth factor, Interleukin 5, leading to increased eosinophil numbers in circulation and in tissues following AS [16,20].

The mast cell secretory products, histamine and eosinophil chemotactic factor of AS, are both strong chemotactic factors for eosinophils [21], leading to heavy infiltration of eosinophils into the lung, small bowel and spleen, as we have observed (Figure 3). The prominent perivascular edema of AS in our rat model may reflect the release of histamine and other vasoactive peptides that can increase vascular permeability, as noted previously [12,13,14,18,19,20,21,22,23,24]. 

The heart shows no remarkable morphological changes evident by hematoxylin–eosin staining in either cardiomyocytes or coronary vessels. In fact, 45 min post-induction of anaphylaxis may not suffice to produce morphologic changes in the heart, whose compact and dynamic muscular nature may minimize or delay the interstitial edema associated with the mast cell degranulation. Those functional and molecular changes that might be expected [12] were undetected in our study. 

Cardiac mast cells are observed in perivascular spaces, between cardiac myocytes and in the adventitia, as noted by Marone et al. in cardiac transplants [25]. 

Marone et al. have studied mast cells in the heart and concluded that human heart mast cells and their mediators play a role in severe AS reactions [25,26,27]. However, mast cells are not as prominent in heart as in lung, small bowel and spleen in our rat model.

The kidneys show features of acute tubular injury, possibly due to prerenal ischemia secondary to severe hypotension associated with AS [28,29], but contributions of vascular hemostasis and sterile inflammatory mediators are also possible. 

The absence of evident hepatic morphologic changes may reflect the dual blood supply of the liver via the portal vein and hepatic artery. The small bowel shows sloughing of the villi of the surface epithelium, likely reflecting ischemia associated with severe hypotension. However, secretory granule release by the abundant mast cell and eosinophil populations in small bowel villi can lead to local villous edema as well as systemic effects [30]. 

Splenic red pulp expansion in the AS group likely reflects expansion of the sinusoids by extravagated fluid and red blood cells, secondary release of vasoactive mediators from mast cells, eosinophils and basophils, and accumulation of mast cells. However, eosinophils, basophils and macrophages may also contribute during the course of anaphylaxis [30,31].

We identified AS-associated increases in eNOS expression in vascular endothelial cells of the lung, heart, and small bowel wall, and increased iNOS expression in bronchial epithelial cells, mast cells and macrophages. We believe that AS-associated tissue infiltration by mast cells represents a major source of NO production, with consequent vasodilation and hypotension contributing to morbidity and mortality [32,33,34,35]. 

A potential limitation of our study was our inability to demonstrate morphologic changes in the heart post-anaphylaxis by light microscopy. Molecular techniques and electron microscopy might be helpful in providing higher resolution in future studies of AS cardiac tissues. 

## 4. Materials and Methods

### 4.1. Ethical Approval 

This study was reviewed and approved by the Institutional Review Board of the United Arab Emirates University, College of Medicine and Health Sciences, Al Ain, Abu Dhabi, United Arab Emirates, Application number: A5/09. Experiments were performed in accordance with protocols approved by the Institutional Animal Care and Research Advisory Committee. 

### 4.2. Animals and Immunization and Treatment Protocol

Male Wistar rats (250 ± 15 g body weight, 4 weeks of age) were housed in groups of 4 in polypropylene cages at 24–26 °C (12 h light-dark cycle, ad libitum food and water), with 1 week’s acclimation to these conditions before experimental manipulation. Two groups of 6 rats were studied, an allergic group and a nonallergic control group. OVA (1 mg) and 3.5 mg aluminum hydroxide adjuvant were homogeneously suspended in 1 mL 0.9% sterile normal saline. Rats in the allergic group were injected subcutaneously (1 mL) below the scapulae with OVA in adjuvant on days 0, 5, and 14. Nonallergic control rats received only adjuvant suspension on the same schedule. AS was induced in allergic group rats by intravenous bolus injection of 1 mg of OVA (14,15). The nonallergic control group received the same injection. Rats of both groups were continuously perfused intravenously with normal saline at 2 mL/h following induction of AS.

### 4.3. Induction and Monitoring of Anaphylactic Shock

One week after the last immunization, rats (7 wk. old) were anesthetized with pentobarbital sodium solution (62.5 mg/kg) administered intraperitoneally. The tracheas were surgically cannulated for artificial ventilation through an endotracheal tube attached to a constant-volume ventilator (Harvard Apparatus, Edenbridge, United Kingdom) set at 60 breaths/min, 6 mL/kg tidal volume, and 5 cm H_2_O end-expiratory pressure at 100% inspired oxygen, at 37 °C (thermo-blanket; Harvard Apparatus, South Natick, MA, USA). A left jugular vein PE10 catheter was for IV treatment via Harvard pump (Harvard Apparatus, South Natick, MA, USA). A left carotid artery catheter was connected via pressure transducer to the Power-Lab blood pressure module (AD Instruments, Bella Vista, NSW, Australia) to measure systolic, diastolic, and MAP and heart rate HR. (Unilateral carotid artery ligation has no detectable effect on cerebral blood flow). Normal saline (0.9%) was infused IV at 2 mL/hr to compensate for intraoperative fluid loss. After the surgical procedures and a subsequent 30-min stabilization period, monitoring hemodynamic parameters at 5 min intervals. For ovalbumin challenge, 1 mg ovalbumin (in 1 mL) was administered IV followed by 60 min infusion of normal saline, with hemodynamic parameter recording at one minute intervals (13,14). 

### 4.4. Tissue Processing

Lungs, hearts, livers, kidneys, small bowels, and spleens were excised, washed with ice-cold saline, blotted with filter paper, weighed and immersion-fixed in 10% buffered formalin. Each organ was sectioned, cassetted and dehydrated in increasing concentrations of ethanol, cleared with xylene and embedded in paraffin. 

Three μM sections cut from paraffin blocks were stained with haematoxylin and eosin and with Sirius red following standard protocols. The stained sections were evaluated by light microscopy by a trained histopathologist. 

### 4.5. Immunohistochemistry

Five μM sections were mounted on aminopropyltriethoxysilane (APES)-coated slides, deparaffinized with xylene, rehydrated in graded alcohol solutions, then subjected to epitope unmasking in 0.01 M citrate (pH = 6.0) at 95 °C for 30 min. Sections were treated with 3% hydrogen peroxide for 15 min to block neutralize endogenous peroxidases, followed by protein block for 60 min. Sections were incubated for one hour at room temperature with either antitryptase (Rabbit monoclonal, clone EP269, 1:100, Cell Marque, USA), anti-c-kit antibody (Rabbit Polyclonal, 1:100, DAKO, Agilent, Santa Clara, California, USA), anti-Nitric oxide synthase, inducible (iNOS), (Rabbit Polyclonal, 1:100, ThermoFisher, Waltham, MA, USA) or anti-eNOS (Rabbit Polyclonal, 1:200, ThermoFisher, Waltham, MA, USA). 

Sections were then incubated with secondary antibody (EnVisionTM Detection System DAKO, Agilent, Santa Clara, California, USA), for 20 min at room temperature, followed by addition of DAB chromogen (EnVisionTM Detection System, DAKO, Agilent, Santa Clara, California, USA), with hematoxylin counterstaining. Omission of primary antibody served as negative controls. Positive and negative controls were used for every experimental batch of slides.

### 4.6. Morphometric Analysis

Morphometric analysis of Sirius red staining of eosinophils and tryptase staining of mast cells was done using ImageJ software (http://rsbweb.nih.gov/ij/). Sirius red and tryptase labeling were determined by counting the number of positive cells in randomly selected 50 high power fields (HPF) in lung, small bowel, and spleen, heart, kidney and liver sections. Numbers of positive cells were quantitated per mm^2^ (Each mm^2^ = 4HPF) [36]. Positive staining of eosinophils with Sirius red was determined by the presence of an orange-to-red granular staining pattern. Tryptase-positive cells were determined by the presence of a brown granular cytoplasmic staining pattern. 

### 4.7. Statistical Analysis

All statistical analyses were performed using GraphPad Prism Software version 5. Comparisons between the various groups were achieved by one-way analysis of variance (ANOVA), followed by Tukey post-hoc tests. *p*-values < 0.05 were considered significant.

## 5. Conclusions

Cellular and immunohistochemical changes in AS are predominantly characterized by accumulation of mast cells and eosinophils in lungs, small bowel, and spleen, rather than in the heart. Targeting NO production may help reduce the morbidity and mortality associated with AS.

## Supplementary Materials

The following are available online at https://www.mdpi.com/2218-273X/9/3/101/s1, Figure S1–Figure S6.
