# Supplementary material for: Cellular and Immunohistochemical Changes in Anaphylactic Shock Induced in the Ovalbumin-Sensitized Wistar Rat Model
Source: Biomolecules. 2019 Mar 13;9(3):101. doi: 10.3390/biom9030101 (PMC6468713; doi:10.3390/biom9030101)
Supplement: Supplementary file 1 [file biomolecules-09-00101-s001.pdf]

biomolecules

Supplementary Materials

Cellular and Immunohistochemical Changes in Anaphylactic Shock Induced in the Ovalbumin-Sensitized Wistar Rat Model

Suhail Al-Salam [1], Elhadi H. Aburawi [2], Suleiman Al-Hammadi [2], Sekhar Dhanasekaran [3], Mohamed Shafiuallah [4], Javed Yasin [5], Manjusha Sudhadevi [1], Aktham Awwad [6], Seth L. Alper [7], Elsadig E. Kazzam [5], and Abdelouahab Bellou [8,9,10,*]

1 Department of Pathology, College of Medicine & Health Sciences, United Arab Emirates University, 17666 AlAin, Abu Dhabi, UAE; suhaila@uaeu.ac.ae (S.A.-S.); m.sudhadevi@uaeu.ac.ae (M.S.)
2 Department of Paediatrics, College of Medicine & Health Sciences, United Arab Emirates University, 17666 AlAin, Abu Dhabi, UAE; E.aburawi@uaeu.ac.ae (E.H.A.); suleiman.alhammadi@uaeu.ac.ae (S.-A.H.)
3 Renaissance LLC, South Brunswick Dayton, New Jersey 08810, USA; sdsekar@outlook.com
4 Department of Pharmacology, College of Medicine & Health Sciences, United Arab Emirates University, 17666 AlAin, Abu Dhabi, UAE; m.shafiullah@uaeu.ac.ae
5 Department of Internal Medicine, College of Medicine & Health Sciences, United Arab Emirates University, 17666 AlAin, Abu Dhabi, UAE; javed.yasin@uaeu.ac.ae (J.Y.); e.kazzam@hotmail.com (E.E.K.)
6 Department of Laboratory Medicine, Tawam Hospital, 15258 AlAin, Abu Dhabi, UAE; aadnan@seha.ae
7 Division of Nephrology and Vascular Biology Research Center, Beth Israel Deaconess Medical Center, Department of Medicine, Harvard Medical School, Boston, MA 02215, USA; salper@bidmc.harvard.edu
8 Department of Emergency Medicine, Beth Israel Deaconess Medical Center, Harvard Medical School, Boston, MA 02215, USA
9 Global HealthCare Network & Research Innovation Institute, Brookline, MA 02446, USA
10 International Board of Medicine and Surgery, Tampa, FL 34677, USA.
* Correspondence: abellou402@gmail.com; Tel.: +161-766-93-707

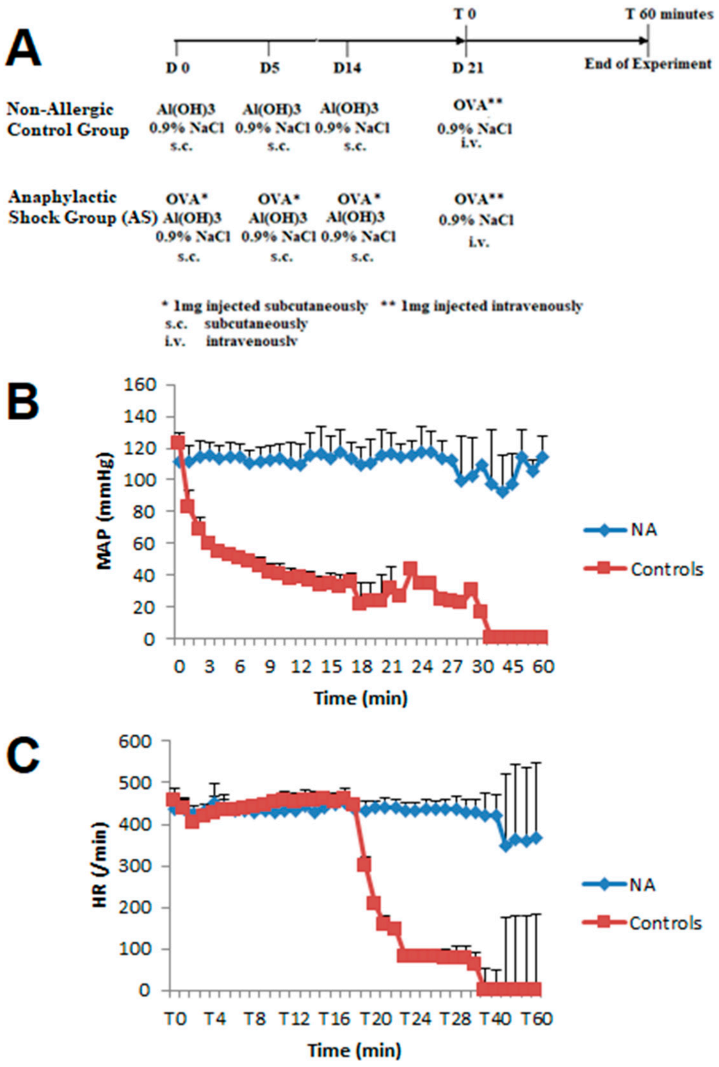

Figure S1. (**A**) Design of the experiment. Rats were sensitized with ovalbumin (OVA) by three intradermal (i.d) injections on day 0 (D0), day 5 (D5), and day 14 (D14). On day 21 (D21), rats were prepared for hemodynamic study by recording mean arterial blood pressure (MAP, **B**) and heart rate (HR, **C**) through a carotid artery catheter placed after anaesthesia. Anaphylactic shock was induced by an i.v. bolus of OVA administered via jugular vein catheter. Hemodynamic parameters (MAP, HR) were recorded every minute for 30 minutes, and then every 5 minutes for 30 additional minutes. Total duration of the experiment post-OVA administration was 60 minutes. Adapted from Bellou et al. (See ref 13).

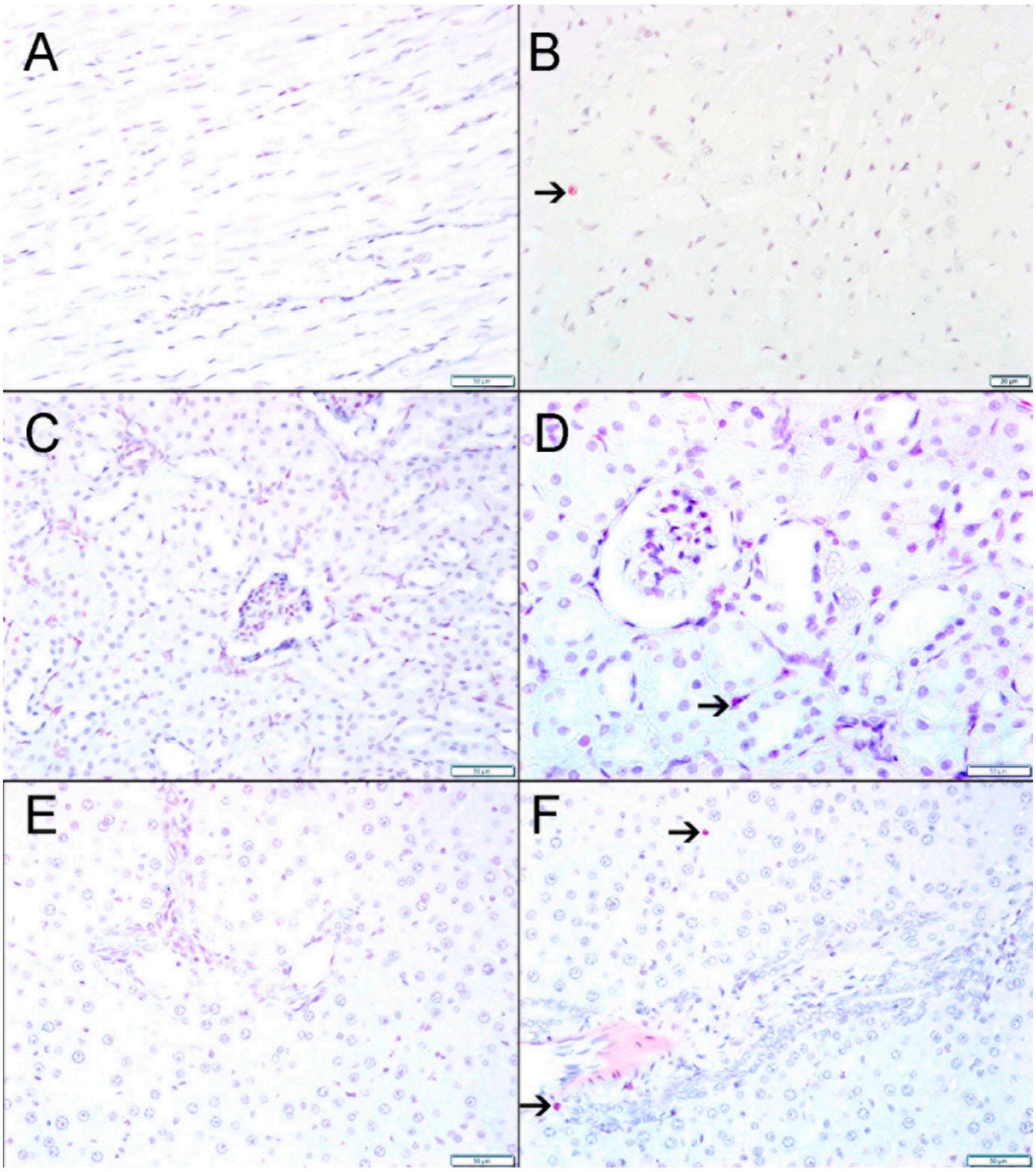

Figure S2. A,C,and **E**: Representative sections from heart (**A**) kidney (**C**)and liver (**E**) of non-allergic control group where eosinophils are not detected**. B,D** and **F**: Representative sections from heart (**B**), kidney (**D**), and liver (**F**) of AS group showing few eosinophils (arrows).

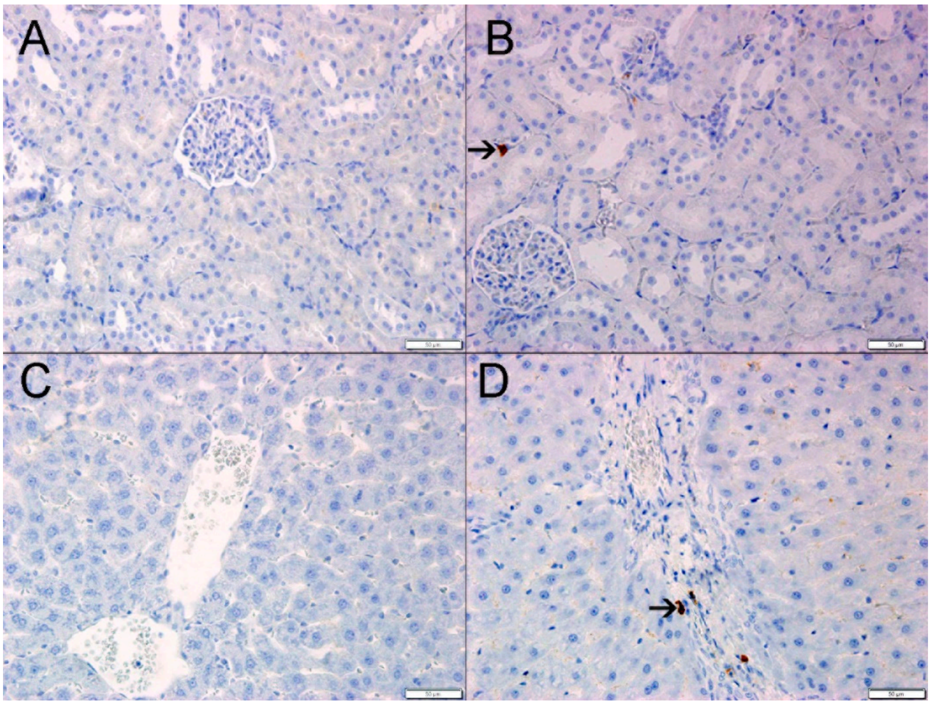

Figure S3. A,C: Representative sections from kidney (**A**) and liver (**C**) of non-allergic control group where tryptase-positive mast cells are not detected. **B,D**: Representative sections from kidney (**B**) and liver (**D**) of As group showing few tryptase-positive mast cells (arrows).

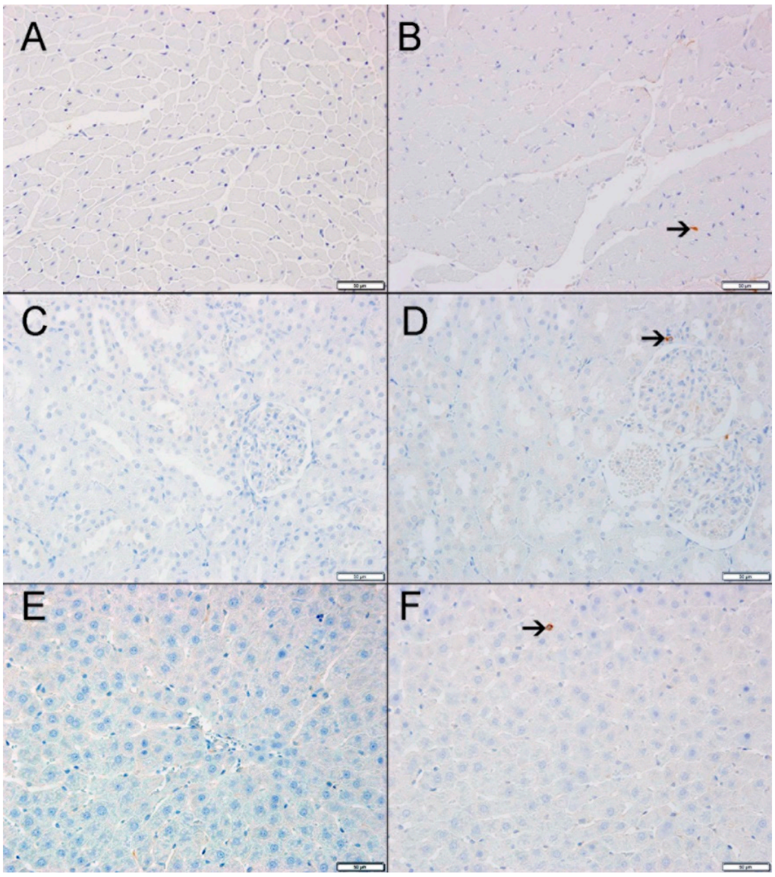

Figure S4. A,C,and **E**: Representative sections from heart (**A**) kidney (**C**) and liver (**E**) of non-allergic control group where c-KIT-positive mast cells are not detected. **B,D** and **F**: Representative sections from heart (**B**), kidney (**D**), and liver (**F**) of AS group showing few c-KIT-positive mast cells (arrows).

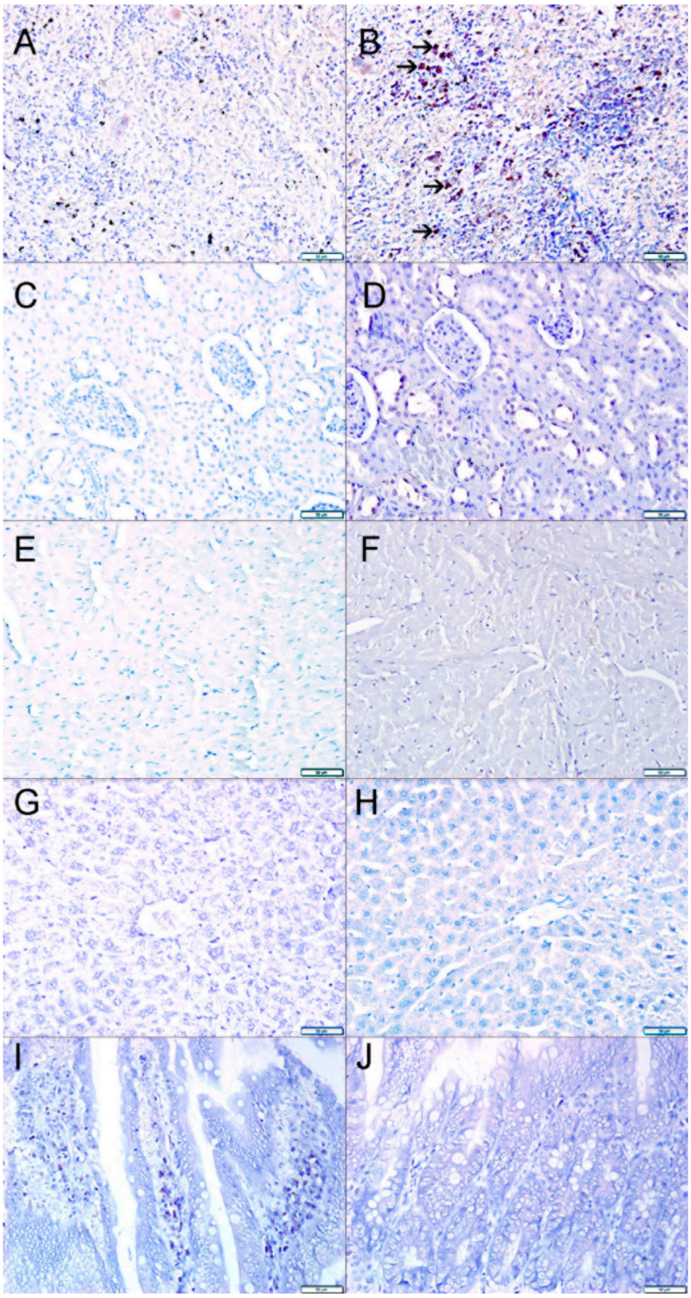

Figure S5. A,C,E,G,I: Representative sections from spleen (**A**), kidney (**C**), heart (**E**), liver (**G**) and small bowel (**I**) of non-allergic control group where iNOS-positive cells are not detected. (**B**): Representative section from spleen of AS group showing few iNOS-positive cells (arrows). **D,F,H** and **J**: Representative sections kidney (**D**), heart (**F**), liver (**H**) and small bowel (**J**) of AS group where iNOS-positive cells are not detected.

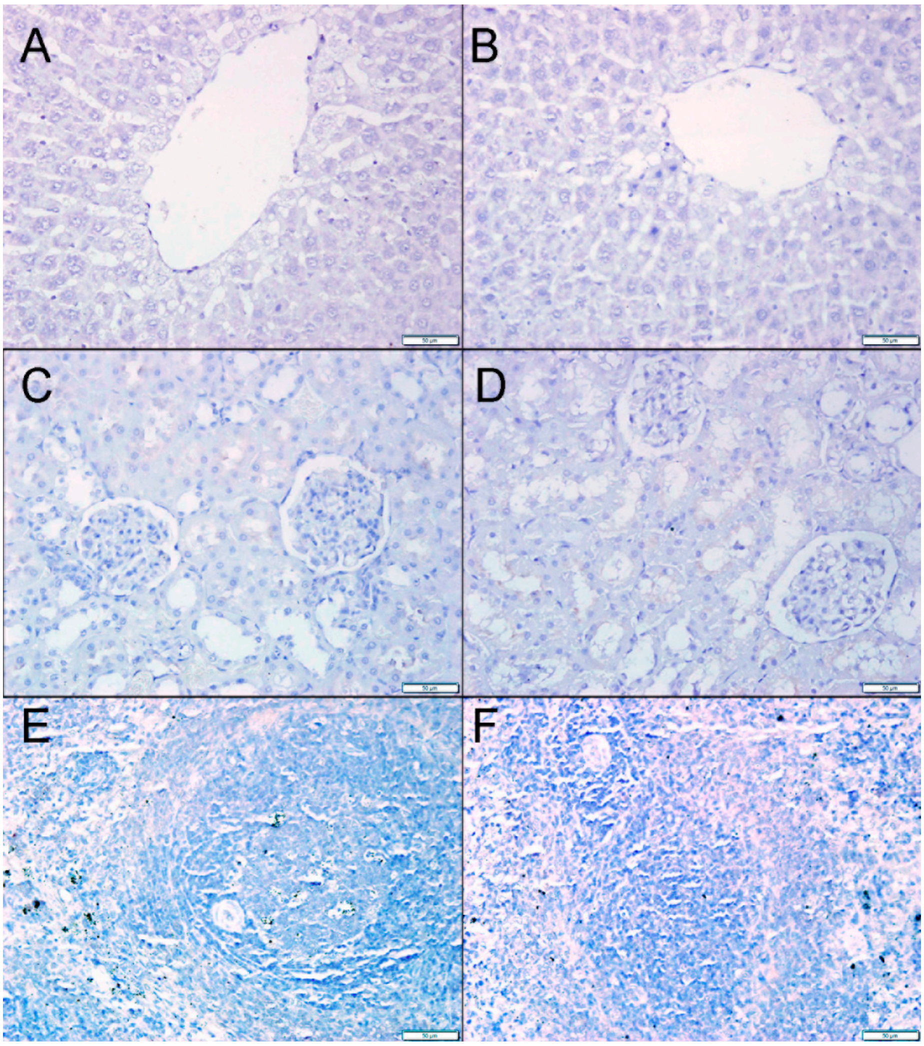

Figure S6. A,C,E: Representative sections from liver (A), kidney (**C**), spleen (**E**) of non-allergic control group where eNOS-positive cells are not detected. **B,D,F**: Representative sections liver (**B**), kidney (**D**), spleen (**F**) of AS group where iNOS-positive cells are not detected.
